# Investigating the activity of Ca_2_Fe_2_O_5_ additives on the thermochemical energy storage performance of limestone waste†

**DOI:** 10.1039/d3ra05875a

**Published:** 2023-11-03

**Authors:** Rehan Anwar, Rajani K. Vijayaraghavan, Patrick J. McNally, Maria Myrto Dardavila, Epaminondas Voutsas, M. Veronica Sofianos

**Affiliations:** a School of Chemical and Bioprocess Engineering, University College Dublin Belfield Dublin 4 Ireland mvsofianou@gmail.com; b School of Electronic Engineering, Dublin City University Glasnevin Dublin 9 Ireland; c School of Chemical Engineering, National Technical University of Athens 9 Iroon Polytechniou Str. 15780 Athens Greece

## Abstract

Efficient and reliable energy storage systems are necessary to address the intermittency and variability of renewable energy sources. Thermochemical energy storage (TCES) has emerged as a promising solution for long-term renewable energy storage, with limestone being a widely studied material due to its abundance and high energy density. However, the practical implementation of limestone-based TCES systems faces challenges related to performance degradation upon multiple energy storage/release cycles, impacting their long-term viability and efficiency. In this study, we investigate the activity of Ca_2_Fe_2_O_5_ additives on the thermochemical energy storage performance of limestone waste. Ca_2_Fe_2_O_5_ additives were synthesized by a wet precipitation method using three different Ca/Fe molar ratios and added to limestone waste in a 5, 10, and 20 weight concentration. The synthesized samples were characterized using XRD, SEM, EDS, BET, and XPS techniques. The thermal properties and heat storage performance of the samples were evaluated through thermogravimetric analysis of calcination/carbonation cycling experiments. The results demonstrate the potential of Ca_2_Fe_2_O_5_ additives to improve the cycling stability and energy storage density of limestone-based TCES systems. The sample with 5 wt% of Ca_2_Fe_2_O_5_ additive having Ca : Fe molar ratio of 1 : 1 outperformed all samples with an effective conversion rate of 0.21 after 40 cycles, 1.31 times higher than limestone waste.

## Abstract 2

Activation energy for CaCO_3_ calcination reaction was reduced using Ca_2_Fe_2_O_5_ additives which improved the thermochemical cycling capacity of limestone waste.

## Graphical abstract

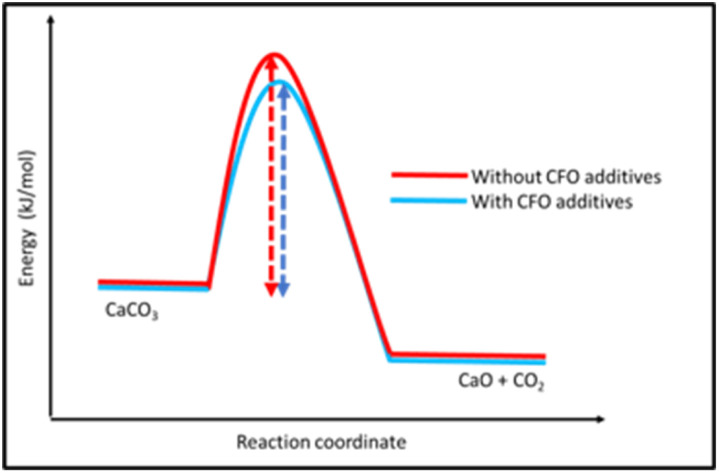

## Introduction

The integration of renewable energy into the electricity grid, necessitates efficient and reliable energy storage systems that can mitigate its intermittency and variability.^1,2^ The most popular energy storage systems reported in the literature are: mechanical storage (*i.e.* pumped hydro storage),^3^ electrochemical storage (*i.e.* Li-ion batteries),^4^ electrical storage (*i.e.* capacitors),^5^ chemical storage (*i.e.* H_2_ storage),^6^ and thermal energy storage (*i.e.* molten salts, phase change materials, metal carbonates).^7^ Thermal energy storage is classified into three categories based on the mode of which heat is being stored, namely sensible heat storage (SHS),^8^ latent heat storage (LHS),^9^ and thermochemical energy storage (TCES).^10^

Thermochemical energy storage (TCES) has emerged as a promising system for long-term renewable energy storage, enabling the efficient conversion and storage of thermal energy.^11^ Among the various TCES materials explored, limestone has attracted considerable attention due to its abundance, low cost, and high energy density (>1000 kJ kg^−1^).^12^ The reversible calcination–carbonation reaction of limestone offers a viable pathway for storing (calcination reaction) and releasing (carbonation reaction) thermal energy.^13,14^ However, the practical implementation of limestone-based TCES systems faces challenges related to performance degradation upon thermal cycling (calcination/carbonation reactions), impacting their long-term viability and efficiency.^15,16^CaCO_3_ ↔ CaO + CO_2_ Δ*H*_860°C_ = 165.9 kJwhere Δ*H*_860°C_ is the theoretically calculated enthalpy of the reaction for 860 °C.

In detail, as thermal cycling progresses, limestone particles undergo agglomeration, sintering, and pore closure, resulting in the decrease of their reactive surface area and available active sites, leading to reactivity losses.^17,18^ Addressing this issue of reactivity losses is critical for enhancing the thermal cycling performance and overall effectiveness of limestone-based TCES systems. In detail, researchers have explored various strategies, with some focusing on the CaCO_3_/CaO particle size optimization,^19–22^ and others on the introduction of inert additives/dopants,^23–26^ and hydration.^27–29^ All these strategies aim to maximize the contact area of limestone particles with the reactant gases, promote efficient heat transfer, and optimize reaction kinetics, thereby improving the overall cycling performance.^30^

The introduction of inert additives/dopants is considered a promising solution to address reactivity losses of limestone particles upon multiple thermal cycles.^31^ Additives can play a crucial role in providing a stable framework that prevents limestone particle sintering, increases their reactive surface area, and mitigates degradation.^32^ A number of additives have been reported in the literature for improving the cyclability of limestone, including Al_2_O_3_,^33–35^ ZrO_2_,^36–39^ and SiO_2_.^40–42^ Table S1† shows the list of additives along with their percentages used, operating temperatures, and product formed. Although these additives improved the cycling performance of limestone, some of its storage capacity was lost due to its reaction with these additives to form new ternary oxides.

Having this in mind, it is essential to use additives that will not only improve the cycling performance of limestone but also will stay inert upon cycling preventing the formation of a new ternary oxide. As such, this study investigates for the first time the activity of Ca_2_Fe_2_O_5_ additives on the thermochemical energy storage performance of limestone waste. Ca_2_Fe_2_O_5_ is an environmentally safe, chemically stable, inexpensive, and abundant perovskite.^43^ A large number of oxygen vacancies present in Ca_2_Fe_2_O_5_ lattice are responsible for its good high ionic conductivity and catalytic activity.^44^ Due to the perovskite properties of Ca_2_Fe_2_O_5_, it has been used for several energy applications such as electrochemical batteries,^45,46^ fuel cells,^47^ supercapacitors, and H_2_ production.^48,49^ The Ca_2_Fe_2_O_5_ additives for this work were synthesized by a wet precipitation method using three different Ca/Fe molar ratios as to tailor the oxygen vacancies in their crystal structure, and were then added to limestone waste in 5, 10 and 20 wt% for preparation of thermal batteries.

## Experimental

### Synthesis of Ca_2_Fe_2_O_5_

Ca_2_Fe_2_O_5_ was synthesized by a simple one-pot wet precipitation method. Three unique samples of Ca_2_Fe_2_O_5_ were prepared by using three different molar ratios of Ca to Fe as shown in Table 1.

**Table 1:** Ca_2_Fe_2_O_5_ sample IDs and stoichiometric ratios

Sample IDs	Stoichiometric molar ratio Ca/Fe
CFO-I	1/1
CFO-II	1/1.05
CFO-III	1.05/1

**Fig. 1:**
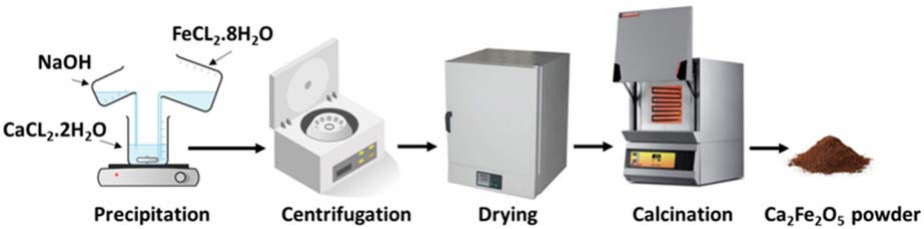
Illustration of the synthesis method for the Ca_2_Fe_2_O_5_ additives.

For the 1/1 stoichiometric molar ratio of Ca/Fe, 10 mmol of FeCl_2_·4H_2_O (>99%, Sigma Aldrich) and 10 mmol of CaCl_2_·2H_2_O (>99%, Honeywell) were dissolved in 45 mL ethanol separately and then were combined under continuous stirring to achieve a uniform solution. 0.06 mol of NaOH (>97%, Sigma-Aldrich) was then added slowly in the form of an aqueous solution to the above solution, achieving an overall molarity of 0.5 M of the final solution. The final solution was left at 50 °C for 24 h under constant stirring on a hotplate. The resulting brown precipitates were centrifuged at 5000 rpm for one minute, and washed twice with distilled water and once with ethanol. They were then dried in an oven at 110 °C for 24 h and finally calcined in a furnace at 1000 °C for 4 h with a heating rate of 3 °C min^−1^ (Fig. 1).

### Ca_2_Fe_2_O_5_/limestone sample preparation

The Ca_2_Fe_2_O_5_/limestone samples were prepared by physically mixing 5, 10, and 20 wt% of Ca_2_Fe_2_O_5_ powder to limestone waste (Kilsheelan limestone Quarries, Ireland) using a mortar and pestle (Table 2).

**Table 2:** Ca_2_Fe_2_O_5_/limestone samples and their allocated sample IDs

wt% Ca_2_Fe_2_O_5_ + wt% limestone	Sample IDs
20% CFO-I + 80% limestone	20CFO-I
20% CFO-II + 80% limestone	20CFO-II
20% CFO-III + 80% limestone	20CFO-III
10% CFO-I + 90% limestone	10CFO-I
10% CFO-II + 90% limestone	10CFO-II
10% CFO-III + 90% limestone	10CFO-III
5% CFO-I + 95% limestone	5CFO-I
5% CFO-II + 95% limestone	5CFO-II
5% CFO-III + 95% limestone	5CFO-III

### Materials characterization

All phase observations were carried out by *ex situ* powder X-ray diffraction (XRD) using a Siemens D500 (40 kV, 30 mA) diffractometer with a Cu-Ka radiation (*λ* = 1.5405 Å), in a *2θ* range of 10–80° with a step size of 0.04°, a scan speed of 1 s/step and a rotational speed of 30 rpm.

Morphological observations for all samples were performed using a scanning electron microscope (SEM) (Zeiss Sigma 300). Prior to SEM imaging, a small amount of the powder sample was placed onto a carbon tape and then was coated with a 4 nm thick Pt layer to reduce charging during SEM imaging. Whereas EDS mapping was carried on a Hitachi Regulus 8230 Field Emission Gun Scanning Electron Microscope (FEGSEM) combined with an Oxford Astec 170 EDS and manipulated by the Aztec software.

Specific surface area measurements were undertaken by a Micromeritics Gemini VII system (Micromeritics, Nor-cross, GA, USA) using nitrogen (N_2_) adsorption at 77 K. The Brunauer–Emmett–Teller (BET) multi-point method was used with relative pressures between 0.05 and 0.30 bar. All samples were degassed at 300 °C in nitrogen atmosphere before proceeding with their N_2_ adsorption analysis.

X-ray photoelectron spectroscopy (XPS) was performed to study the chemical state of the Ca_2_Fe_2_O_5_ additives by utilizing a Kratos AXIS Ultra DLD X-ray photoelectron spectrometer with an Al-Kα X-ray source (1486.7 eV) in ultra-high vacuum. The XPS data was analysed by the Casa XPS software, and calibrated using the surface adventitious C 1s peak at 284.5 eV.

20CFO-I, 20CFO-II and 20CFO-III samples were subjected to *in situ* powder X-ray diffraction using a triple-axis Jordan Valley Bede D1 high-resolution XRD system with a copper (*λ* = 1.5405 Å) radiation source ran at 45 kV and 40 mA. An Anton Paar DHS 1100 heating stage was a feature of the Bede D1 system that permitted *in situ* temperature-dependent measurements in a vacuum setting. After being compressed into a pellet with a 20 mm diameter and 2 mm thickness, the powder samples were mounted onto the heating stage. XRD measurements were performed at temperatures ranging from room temperature (RT) to 1000 °C. For heating from RT-500 °C and 800–1000 °C, the rate of heating was 500 °C min^−1^. The heating rate was 20°C min^−1^ for heating from 500 to 800 °C. Each scan took about 13 minutes to complete and used the *θ*–2*θ* configuration to measure angles between 20 and 40° at a step size of 0.1°.

### Thermogravimetric analysis

Simultaneous differential scanning calorimetry and thermogravimetric analysis (DSC-TGA) was carried out on using a Netzsch STA 449 F5 instrument. For thermal analysis of limestone waste, a small quantity of the sample, weighing between 35 and 40 mg, was placed into an alumina crucible. The powdered limestone waste was then heated from room temperature to 1000 °C under a N_2_ flow of 20 mL min^−1^ at a rate of 20 °C min^−1^. After reaching the maximum temperature, the sample was kept isothermal for a duration of 30 minutes under a CO_2_/N_2_ flow of 100 mL min^−1^ and 20 mL min^−1^, respectively. Subsequently, the sample was cooled down to room temperature at a rate of 20 °C min^−1^ under the same atmosphere.

The Kissinger method was used to calculate the activation energy for the calcination reaction using the multiple heat rate thermal analysis measurements (eqn (1)).^50,51^ In detail, the sample weighting 35–40 mg, was heated from room temperature to 1000 °C at heating rates of 5, 10 and 20 °C min^−1^, was then kept isothermal for 30 minutes, and finally cooled down to room temperature. All activation energy measurements were taken under N_2_ flow of 20 mL min^−1^.1
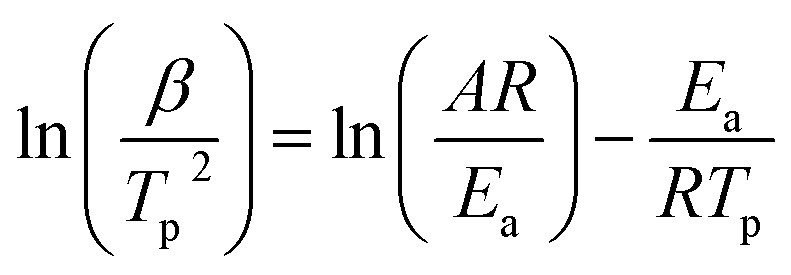
where *β* is heating rate in K min^−1^, *T*_p_ is the DSC peak temperature in K, *E*_a_ is the apparent activation energy of the reaction in J mol^−1^, *R* is the perfect gas constant (8.314 J mol^−1^ K^−1^) and A is the pre-exponential factor (min^−1^). The temperature accuracy (±0.2 K) and sensitivity of DSC (balance accuracy ± 0.1 μg) were calibrated using In, Zn, Al, Ag and Au reference materials.

### Calcination/carbonation cycling and heat storage performance

A thermogravimetric analyser STA 449 F5 manufactured by Netzsch was employed for executing calcination and carbonation cycles at a constant temperature of 860 °C. The calcination process was carried out under a N_2_ flow of 20 mL min^−1^ for 10 minutes, while the carbonation process was performed under a mixture of CO_2_ and N_2_ flows of 100 mL min^−1^ and 20 mL min^−1^, respectively, for 20 minutes. Initially, all samples were heated from room temperature to 860 °C under a N_2_ flow of 20 mL min^−1^ at a heating rate of 20 °C min^−1^. Finally, at the completion of the cycles, all samples were cooled down to room temperature under a CO_2_/N_2_ flow of 100 mL min^−1^ and 20 mL min^−1^, respectively. The final experimental results were the average of the three groups of experimental data to ensure precision and repeatability of the experiment.

The evaluation of energy storage performance of the samples involved the assessment of their effective conversion and energy storage density. The effective conversion, denoted by *X*_ef,N_ was determined as the ratio of the mass of CaO reacted during each carbonation cycle to the total mass of the sample prior to carbonation, as defined by eqn (2). This parameter is a critical indicator of cycling stability and is directly proportional to the energy storage capacity.2
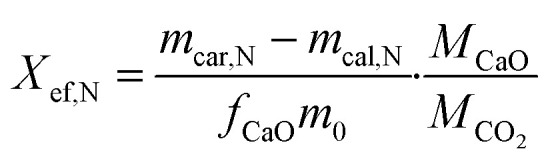
where *N* is the number of calcination/carbonation cycles, *m*_car,N_ and *m*_cal,N_ is the mass of the sample after the *N*th carbonation and *N*th calcination respectively. *m*_o_ is the original mass of the sample including the mass of any additives used, *f*_o_ is the initial fraction of CaO present in the sample, *M*_CaO_ and *M*_CO_2__ represent the molar masses of CaO and CO_2_ in g mol^−1^, respectively.

The maximum amount of heat that can be discharged per unit mass of the samples during each carbonation reaction is represented by the heat storage density, *E*_g,N_ expressed in kJ kg^−1^. This quantity can be calculated using eqn (3), and it serves as an important parameter in characterizing the heat storage performance of the samples.3
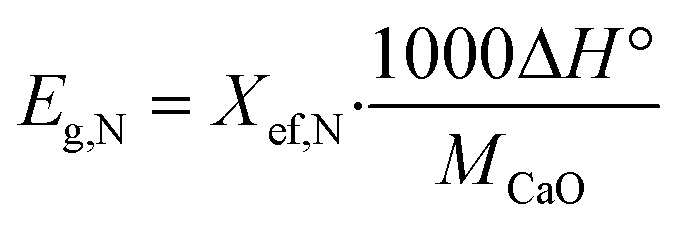
where Δ*H*° is the enthalpy of reaction for 860 °C is equal to 165.9 kJ mol^−1^.

## Results and discussion

**Fig. 2:**
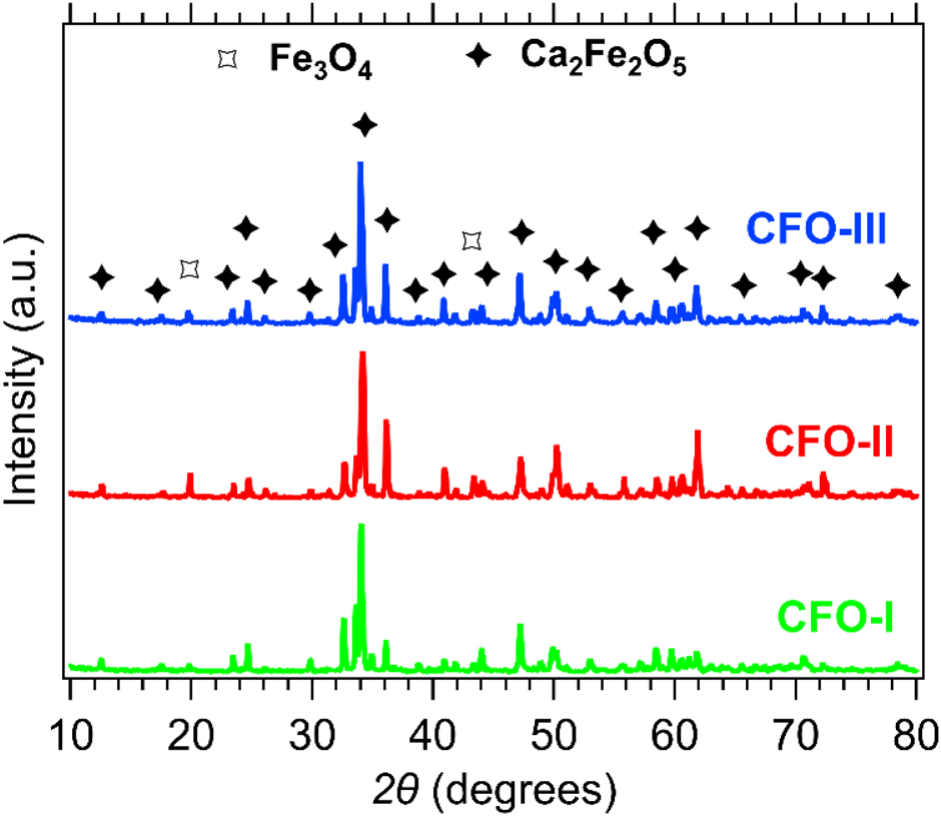
XRD patterns of CFO-I (green), CFO-II (red) and CFO-III (blue).

XRD patterns of the CFO-I, CFO-II and CFO-III samples are shown in Fig. 2. All three samples have the prevalent phase that corresponds to Ca_2_Fe_2_O_5_ (PDF 96-901-4372). Two small peaks identified at 2*θ* values of 20° and 43° correspond to Fe_3_O_4_ (PDF 96-210-7250),^52,53^ a by-product of the wet precipitation method.

**Fig. 3:**
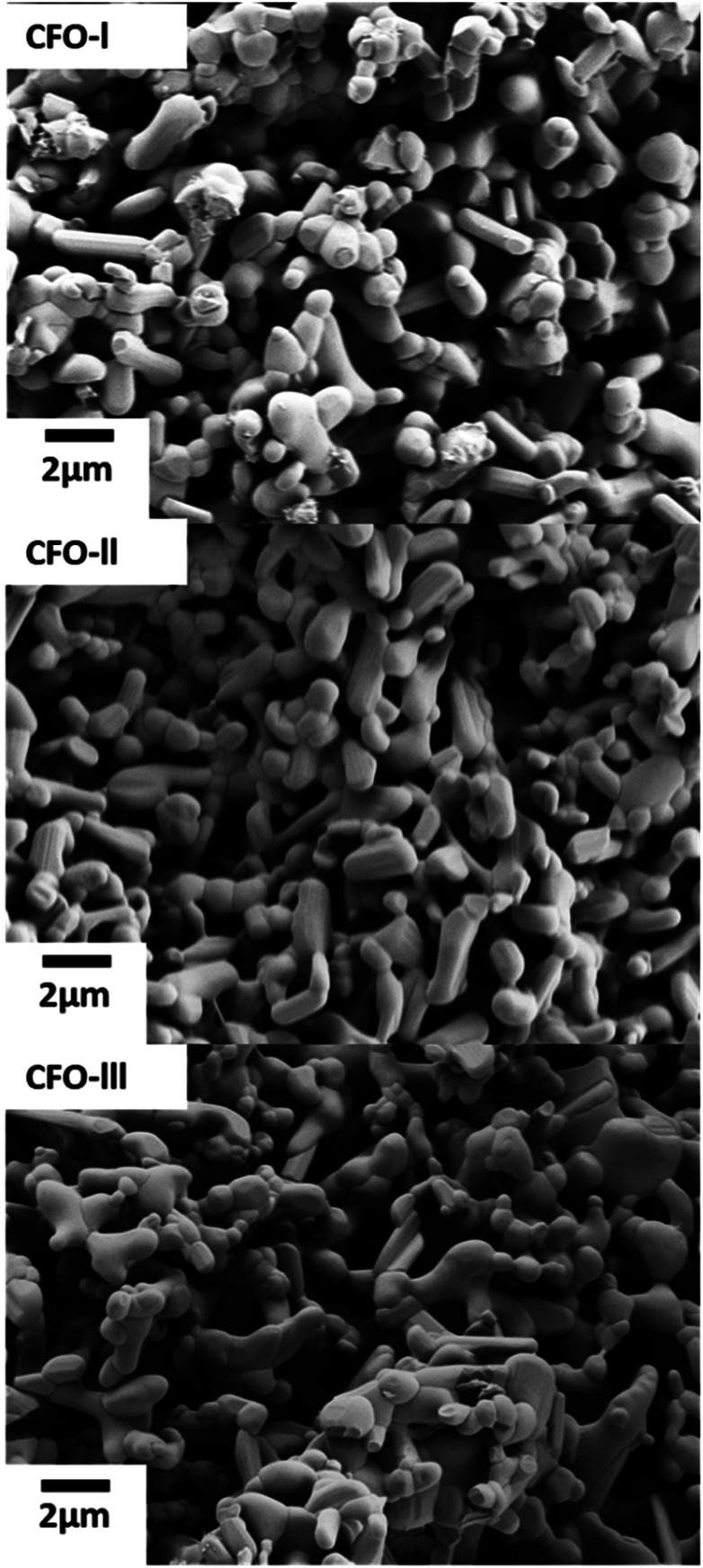
SEM images of CFO-I, CFO-II and CFO-III.

Fig. 3 shows the SEM micrographs of the as-prepared Ca_2_Fe_2_O_5_ samples. All three samples despite having slightly different Ca/Fe molar ratios did not reveal any major change in morphology, there was though a slight change in their average particle size, as was observed. In detail, the average particle size of CFO-I, CFO-II and CFO-III was 1.38, 1.44 and 1.18 μm, respectively (Fig. S10†). The synthesis of pure Ca_2_Fe_2_O_5_ required high calcination temperature (1000 °C), for that reason the synthesized particles are in micro meter range.

BET surface area of CFO-I CFO-II and CFO-III was measured to 2.15, 1.92 and 3.26 m^2^ g^−1^ respectively. CFO-III with the highest Ca/Fe molar ratio exhibited the highest surface area and the lowest average particle size. Whereas CFO-II, with lowest Ca/Fe molar ratio, exhibited the lowest surface area and the highest average particle size. Indicating that one can fine tune the particle size by adjusting the Ca/Fe molar ratio during synthesis.

**Fig. 4:**
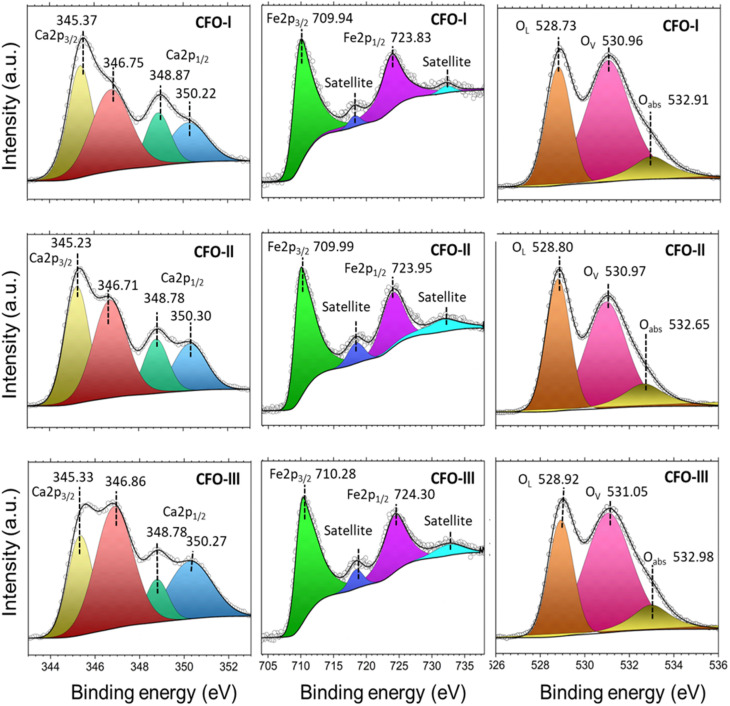
XPS spectra of CFO-I, CFO-II and CFO-III; hollow circle represents the raw intensity while the black solid line represents the peaks fit.

The chemical analysis of Ca_2_Fe_2_O_5_ as-prepared samples is presented in Fig. 4. In detail, the Ca 2p high resolution spectra show four deconvoluted peaks. The two peaks positioned at ∼345.3 eV (Ca 2p_3/2_) and ∼348.8 eV (Ca 2p_1/2_) represent Ca^2+^ from Ca_2_Fe_2_O_5_.^54^ Whereas, the other two peaks at ∼346.8 eV (Ca 2p_3/2_) and ∼350.3 eV (Ca 2p_1/2_) can be related to the presence of CaO traces.^55^ The high resolution Fe 2p spectra was deconvoluted into four peaks which include two satellite peaks. Fe 2p_3/2_ peak at ∼710 eV is associated with Fe^2+^ and Fe 2p_1/2_ peak at ∼724 eV corresponds to Fe^3+^ in Ca_2_Fe_2_O_5_.^54^ By analysing the high-resolution O 1s spectra, it was possible to deconvolute the peaks into three distinct peaks located at approximately 528.8 eV, 531 eV, and 533 eV. These peaks were attributed to different oxygen species, namely oxygen lattice (O_lat_), oxygen vacancy (O_vac_) and the oxygen absorbed (O_abs_) respectively.^56^Table 3 displays the exact binding energy values of Ca 2p, Fe 2p and O 1s for CFO-I, CFO-II and CFO-III. There was no significant shift observed in binging energy values of Ca_2_Fe_2_O_5_ samples with changing the Ca/Fe molar ratio. The percentage (%) of O_vac_ calculated for CFO-I, CFO-II and CFO-III are 58.69, 51.23 and 59.44 respectively. CFO-III with highest Ca/Fe ratio represented the highest percentage O_vac_. Excess Calcium ions might have introduced charge imbalances within the crystal lattice which led to formation of oxygen vacancies to compensate for the excess positive charge introduced by calcium ions. It is expected that higher percentage of O_vac_ will facilitate the ionic movements during calcination and carbonation of limestone.

**Table 3:** Binding energy values (eV) for CFO-I, CFO-II and CFO-III as determined by XPS

Sample name	Ca		Ca		Fe	Fe	O 1s	O 1s	O 1s	O_vac_ (%)
	2p_3/2_		2p_1/2_		2p_3/2_	2p_1/2_	(lat)	(Vac)	(abs)	
CFO-I	345.37	346.75	348.87	350.22	709.94	723.83	528.73	530.96	532.91	58.69
CFO-II	345.23	346.71	348.78	350.3	709.99	723.95	528.8	530.97	532.65	51.23
CFO-Ill	345.33	346.86	348.78	350.27	710.28	724.3	528.92	531.05	532.98	59.44

**Fig. 5:**
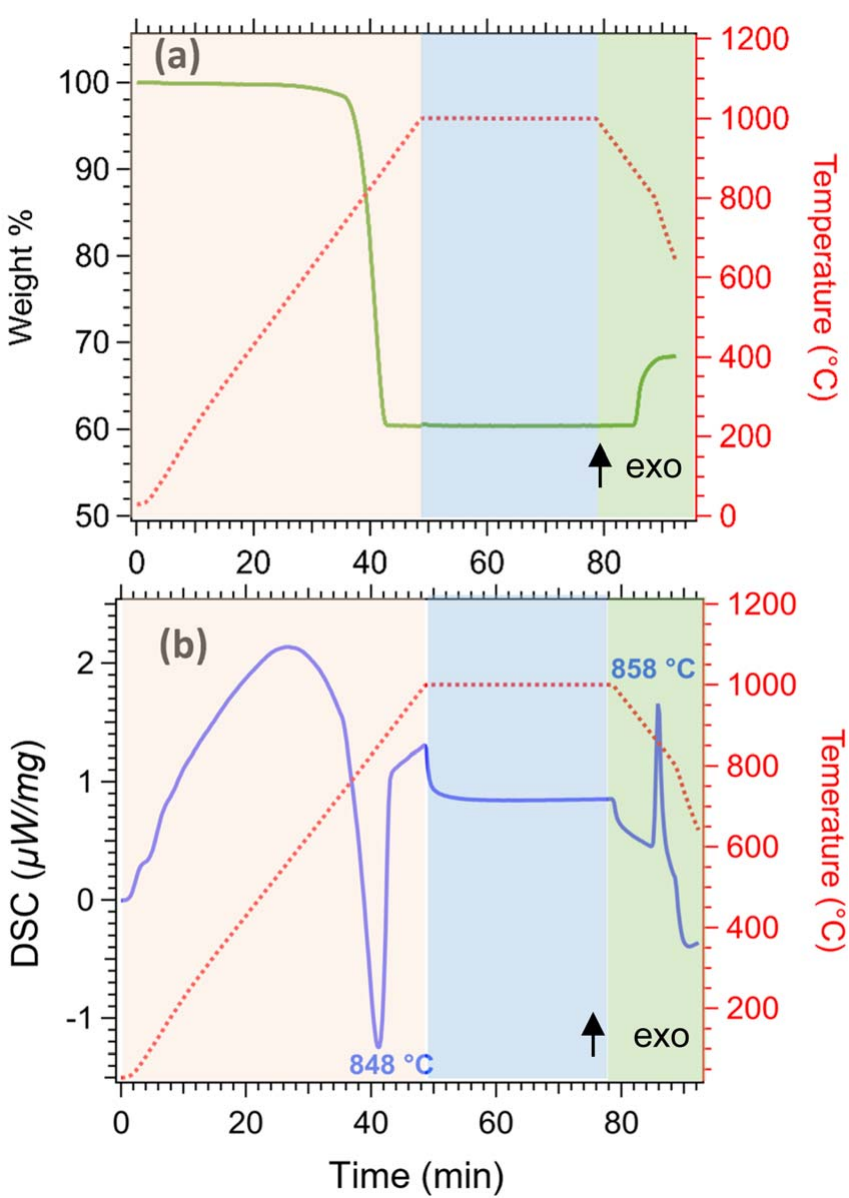
Thermal analysis of limestone waste (a) thermogravimetric analysis (TGA) (b) differential scanning calorimetry (DSC).

Thermal analysis of limestone waste was performed to determine the operating temperature for the cycling experiments, and was conducted in three steps (Fig. 5). In the first step (orange area of Fig. 5), limestone waste was heated from room temperature to 1000 °C under 20 mL min^−1^ N_2_, then was kept isothermal for 30 min under a 20 mL min^−1^ N_2_ and 100 mL min^−1^ CO_2_ atmosphere (second step, blue area of Fig. 5) and finally was cooled down to room temperature in the same atmosphere as the second step (third step, green area in Fig. 5). During the first step, the calcination reaction began at a temperature of 752 °C and reached its peak at 848 °C (Fig. 5b), resulting in a total mass loss of 40 wt% (Fig. 5a). This mass loss is equal to the amount of CO_2_ released during the calcination reaction. The absence of any mass gain during the isothermal period indicates that the temperature was too high for the carbonation reaction to take place (Fig. 5a). While during cooling, the initiation of the carbonation reaction was observed at 872 °C, with a peak maximum at 858 °C (Fig. 5b). The mass gain for this reaction was only equal to 8.06 wt%, which corresponds only to 20% gain of the initial CO_2_ amount released during calcination, representing that the carbonation reaction was incomplete (Fig. 5a).

**Fig. 6:**
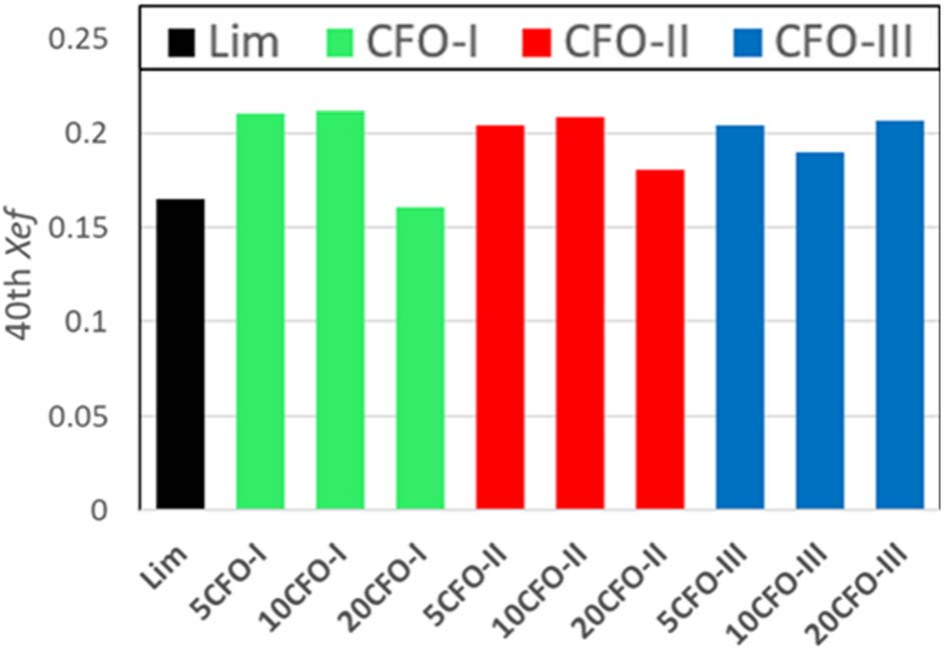
Effective conversion of all samples for the 40th cycle.

The effective conversion of limestone waste and the mixture samples is presented in Fig. 6. The thermochemical performance of limestone waste progressively declined during cycling, resulting in an effective conversion rate of 0.16 at the 40th cycle (Fig. 6 and S2†). Both 5CFO-I and 10CFO-I samples showed similar performance, achieving an effective conversion rate of 0.21 after 40 cycles. In contrast, the 20CFO-I sample had a lower effective conversion rate of 0.16. This, may be attributed to the fact that the higher proportion of CFO-I reduced the exposure of limestone to the reacting gases, resulting in a lower effective conversion.

Likewise, the 5CFO-II and 10CFO-II samples with an effective conversion rate of 0.20 outperformed the 20CFO-II sample with an effective conversion rate of 0.18, after 40 cycles.

On the other hand, both the 5CFO-III and 20CFO-III samples exhibited very similar performance, achieving an effective conversion rate of 0.20 after 40 cycles. However, the 10CFO-III sample showed a slightly lower effective conversion rate of 0.18. The unique behaviour of CFO-III is linked to its highest% oxygen vacancies, highest BET surface area and the lowest average particle size.

Overall, all three additives exhibited the ability to improve the thermochemical cycling performance of limestone waste by increasing the active sites; linked to particle size and oxygen vacancies (Fig. S1†). Each additive possessed its own unique particle size, BET surface area, and oxygen vacancy characteristics, thus requiring an optimum percentage for achieving the best cycling performance. Among all the samples, 5CFO-I stands out as the top performer, having the least additive percentage, exhibiting an effective conversion rate of 0.21, and an energy storage density of 622 kJ kg^−1^ after 40 cycles. This value of energy storage density is comparable to the ZrO_2_/Al_2_O_3_ system^38^ reported in the literature with an energy storage density of 675 kJ kg^−1^, and higher to the ZrO_2_ system^37^ with an energy storage density of 455 kJ kg^−1^. On the other hand, limestone waste showed an effective conversion of 0.16 and an energy storage density of 487 kJ kg^−1^ after 40 cycles (Fig. S2†).

**Fig. 7:**
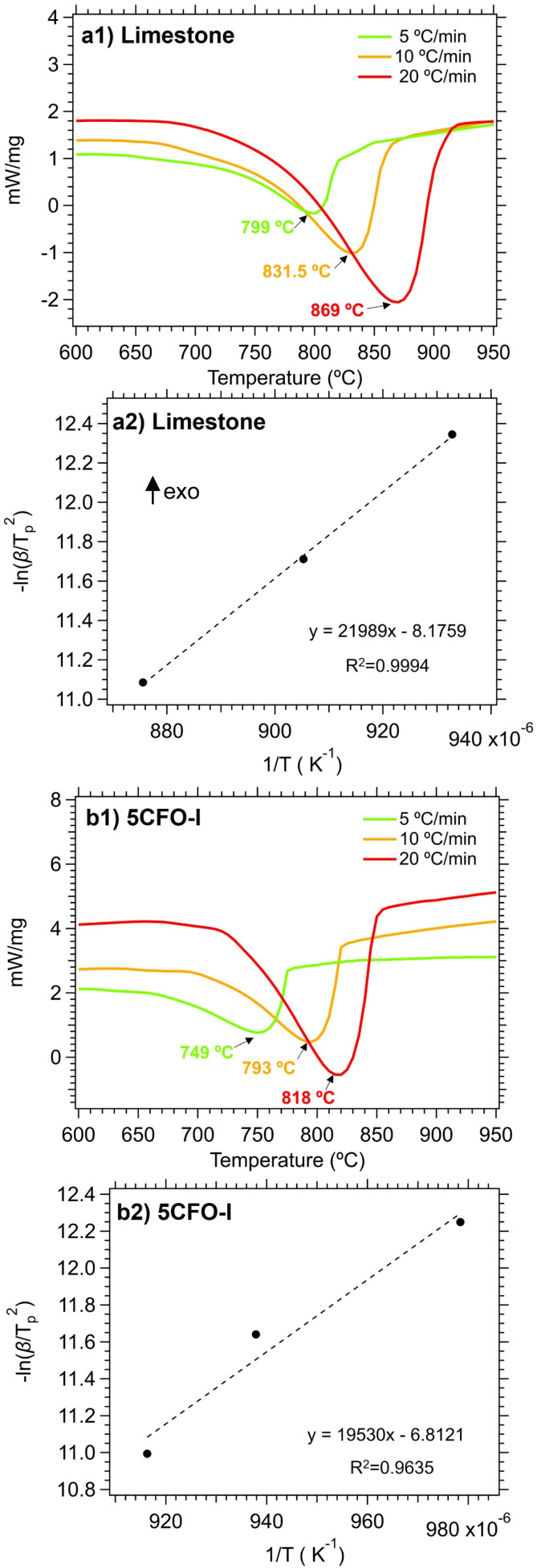
Calcination reaction DSC measurements for limestone waste (a1) and 5CFO-I (b1) at three different heating rates, along with their corresponding Kissinger plots (a2) limestone and (b2) 5CFO-I.

In order to have a better understanding of the additive effect in the calcination reaction, the activation energies of both limestone waste and 5CFO-I were calculated. In detail, Fig. 7 illustrates the DSC measurement of limestone waste and 5CFO-I for the calcination reaction at heating rates of 5, 10 and 20 °C min^−1^ along with their corresponding Kissinger plots. The peak temperatures for the limestone waste were identified as 799 °C, 831.5 °C and 869 °C at heating rates of 5, 10 and 20 °C min^−1^ respectively. Similarly, the peaks temperatures for 5CFO-I were observed as 749 °C, 793 °C and 818 °C at heating rates of 5, 10 and 20 °C min^−1^ correspondingly. The activation energy for the calcination reaction of limestone waste was calculated as 182.8 kJ mol^−1^ and for 5CFO-I as 162.3 kJ mol^−1^. The inclusion of CFO-I additive improved reaction kinetics, as indicated by the lower activation energy of 5CFO-I. This higher effective conversion of 5CFO-I is the result of this improved reaction kinetics.

**Fig. 8:**
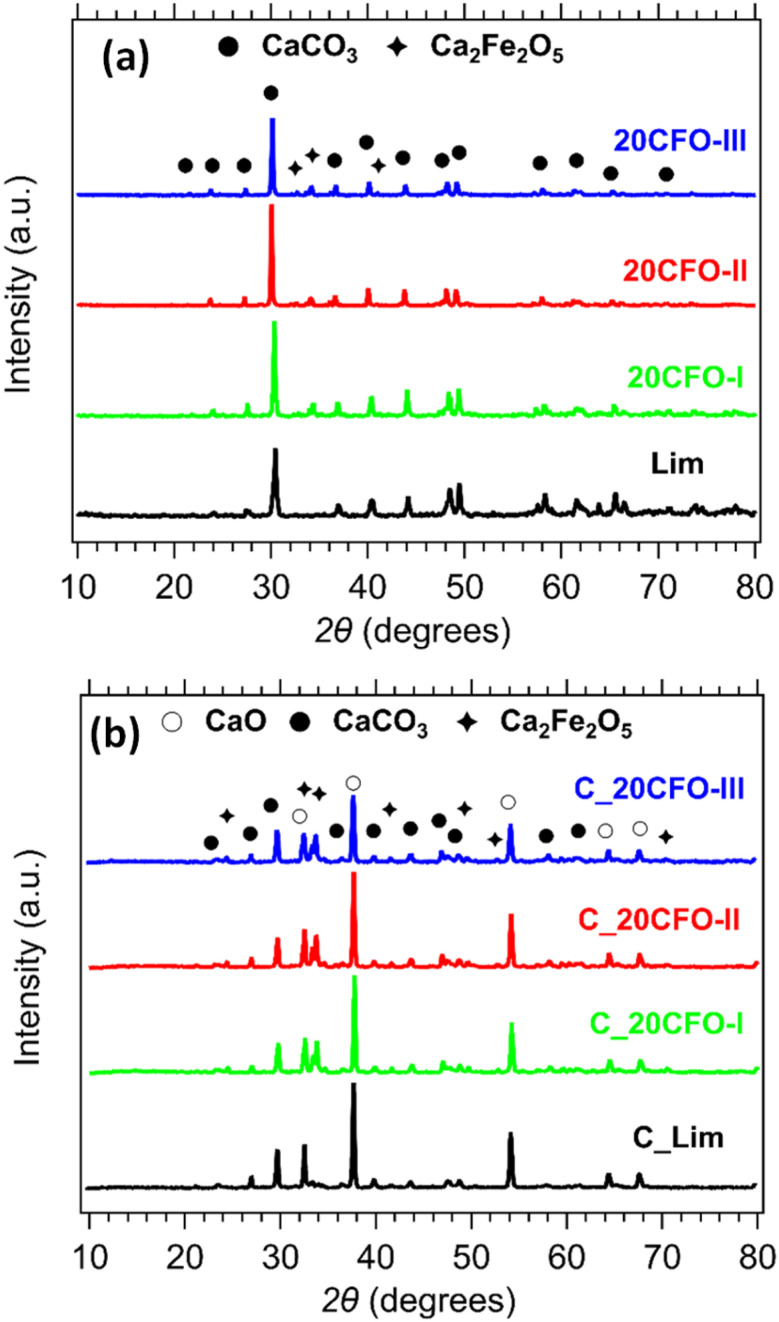
XRD patterns of Lim and 20CFO before (a) and after cycling (b).

The phase composition of the 20CFO samples before and after cycling is presented in Fig. 8. The prevailing crystal phase in all of the as-prepared samples, including the limestone waste, was CaCO_3_, as observed in Fig. 8a (Fig. S3a and S4a†). This suggests that any impurities that might have been present in the limestone waste are at such low levels that they could not be detected through X-ray diffraction. As the amount of Ca_2_Fe_2_O_5_ content increased in the mixture samples, the diffraction peaks assigned to the secondary phase of Ca_2_Fe_2_O_5_ became increasingly apparent, as anticipated. After 40 cycles, CaO is the dominant phase observed in all samples, while CaCO_3_ and Ca_2_Fe_2_O_5_ are also present (Fig. 8b, S3b and S4b†). Indicating that the carbonation reaction was incomplete at the end of the 40th cycle. *In situ* XRDs of 20CFO samples confirmed that no reaction products were observed between CaCO_3_ and Ca_2_Fe_2_O_5_ upon cycling (Fig. S9†). This observation suggests that Ca_2_Fe_2_O_5_ maintained its chemical stability throughout the cycling process, acting as an inert additive that did not form any ternary oxide upon cycling.

**Fig. 9:**
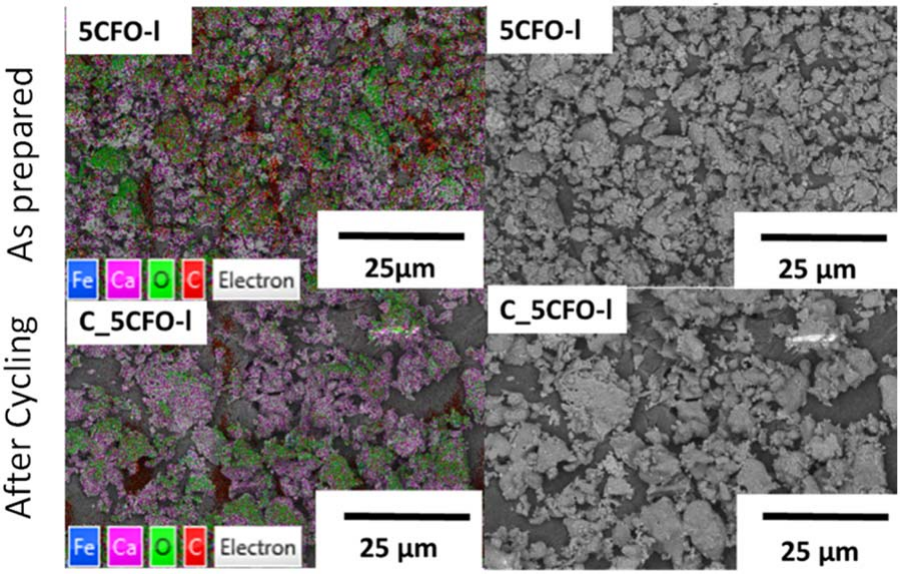
EDS mapping of 5CFO-I as prepared and C_5CFO-I after cycling.

**Fig. 10:**
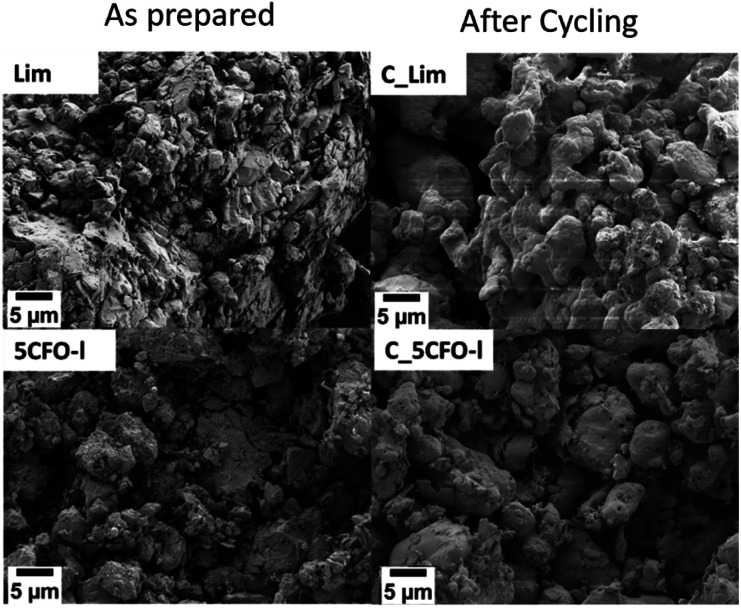
SEM images of 5CFO-I and Lim as prepared and after cycling.

Fig. 9 shows the EDS mapping of 5CFO-I before and after cycling. As the percentage of additive was only 5%, the presence of Fe is not clearly visible (Fig. S5†), but the indication of Fe was clearly seen in 20% samples both before and after cycling (Fig. S6†). In addition, the SEM micrographs of sample 5CFO-I and the limestone waste are shown in Fig. 10. It is evident that the limestone waste particles exhibit significant sintering after cycling, whereas the particles in 5CFO-I display comparatively less sintering (micrographs of all other samples are shown in Fig. S7 and S8†). The fact that less sintering is observed in all mixture samples can be attributed to the Ca_2_Fe_2_O_5_ particles acting as a physical barrier among the limestone particles, preventing them from extended sintering upon cycling as seen for pure limestone waste. It can be concluded that sintering is one of the major causes for reduction in effective conversion of limestone.

## Conclusions

In conclusion, this study explored for the first time the use of Ca_2_Fe_2_O_5_ additives for enhancing the thermochemical energy storage performance of limestone waste. The Ca_2_Fe_2_O_5_ additives were synthesized with three different Ca/Fe molar ratios and added to limestone waste to prepare thermal batteries for long-term renewable energy storage. The mixture samples were morphologically and chemically characterized, and their thermochemical energy storage performance were evaluated through calcination/carbonation cycling experiments. The results indicate that the Ca_2_Fe_2_O_5_ additives can effectively improve the cycling stability and energy storage performance of limestone-based TCES systems. The Ca_2_Fe_2_O_5_ additive did not react with the limestone waste upon cycling to form a ternary oxide; as confirmed from the XRD analysis of the after cycling samples. For all three Ca_2_Fe_2_O_5_ additives (CFO-I, CFO-II and CFO-III), 5 wt% was the most effective additive percentage for multiple thermal cycles. 5CFO-I performed the best, achieving an effective conversion rate of 0.21 after 40 cycles which is 1.31 times better performance than that of limestone waste, having an effective conversion rate of 0.16 after 40 cycles. The optimized Ca/Fe ratio and wt% of additives were found to play a significant role in enhancing the performance of the thermal batteries. This study provides valuable insights into the design and optimization of limestone-based TCES systems, highlighting the potential of Ca_2_Fe_2_O_5_ additives for efficient and reliable long-term energy storage. Further research can focus on optimizing the synthesis process and exploring other additives to further improve the performance of limestone-based TCES systems.

## Conflicts of interest

There are no conflicts to declare.

## Supplementary Material

RA-013-D3RA05875A-s001
