# Supplementary material for: Investigating the activity of Ca2Fe2O5 additives on the thermochemical energy storage performance of limestone waste
Source: RSC Adv. 2023 Nov 3;13(46):32523–31. doi: 10.1039/d3ra05875a (PMC10624235; doi:10.1039/d3ra05875a)
Supplement: RA-013-D3RA05875A-s001 [file RA-013-D3RA05875A-s001.pdf]

Electronic Supplementary Material (ESI) for RSC Advances.
This journal is © The Royal Society of Chemistry 2023

Electronic Supplemental Information for:

Exploring the Influence of $Ca_2Fe_2O_5$ Additives on Thermochemical Energy Storage Capacity of Waste Limestone

Rehan Anwar[a], Rajani K. Vijayaraghavan[b], Patrick J. McNally[b], Maria Myrto Dardavila[a,c], Epaminondas Voutsas[c], M. Veronica Sofianos[a*]

[a]School of Chemical and Bioprocess Engineering, University College Dublin, Belfield, Dublin 4, Ireland

[b]School of Electronic Engineering, Dublin City University, Glasnevin, Dublin 9, Ireland

[c]School of Chemical Engineering, National Technical University of Athens, 9 Iroon Polytechniou Str., 15780 Athens, Greece

E-mail: mvsofianou@gmail.com

Table 1S Description of additive studies for $CaCO_3$ based TCHS

Additive	Wt % additive	Operating Temperature (°C)	Formed Product	Reference
$CaCO_3$ Pure	N/A	750-925	N/A	1
Al_2O_3	2.5	850-900	$Ca_{12}Al_{14}O_{33}$	2
Al_2O_3	16.7	850-950	$Ca_5A_{l6}O_{14}$ $Ca_{12}Al_{14}O3$	3
Al_2O_3	34	850-950	Ca3Al2O6	4
Al_2O_3	13.3	900	Ca5Al6O14	5
Al_2O_3	5	725-850	Ca4Al6O13	6
ZrO_2	34	950	CaZrO3	3
ZrO_2	20	900	CaZrO3	5
ZrO_2	5	850-750	CaZrO3	7
ZrO_2	15	850-750	CaZrO3	7
ZrO_2	30	850-750	CaZrO3	7
ZrO_2	20	900	CaZrO3	8
SiO_2	5	700	-	9
SiO_2	20	900	$Ca_5(SiO_4)_2CO_3$	8
SiC	20	884	-	10
Fe_2O_3	20	900	Fe_3O_4, $Ca_2Fe_2O_5$	8

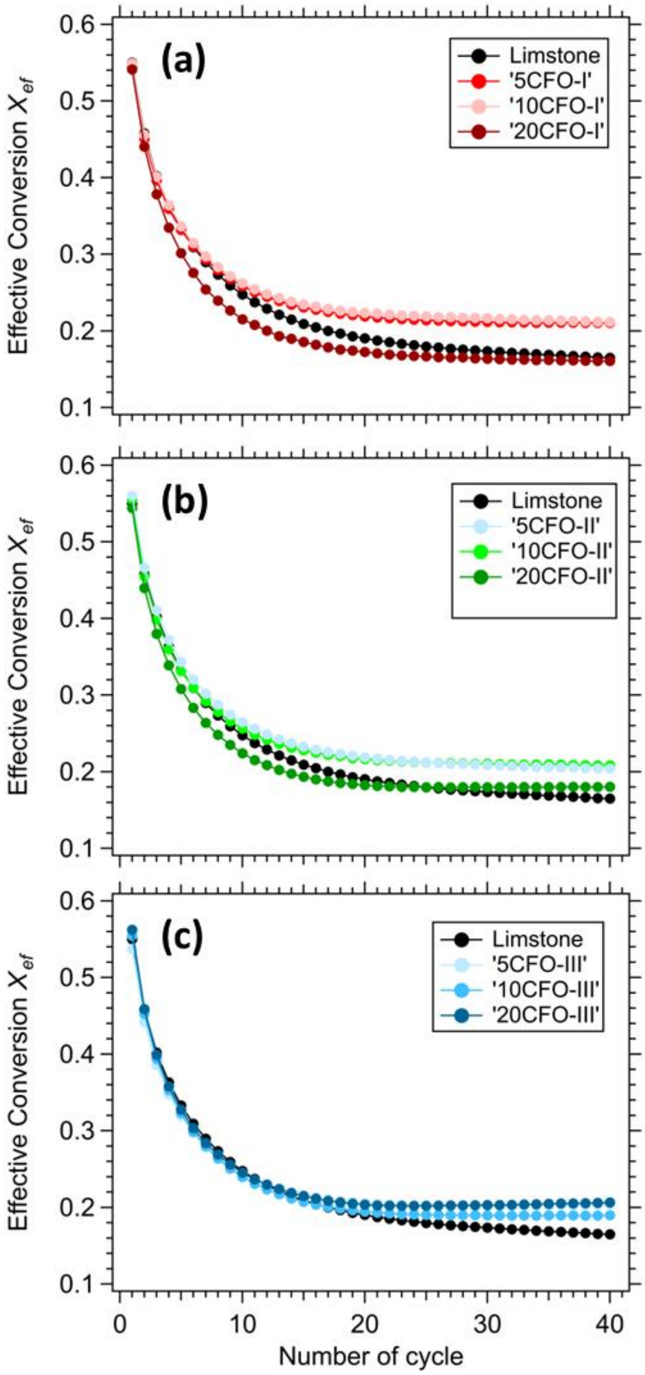

Fig. S1 Effective conversion *vs* number of cycles for 5,10 and 20% additive (a) CFO-I (b) CFO-II (c) CFO-III

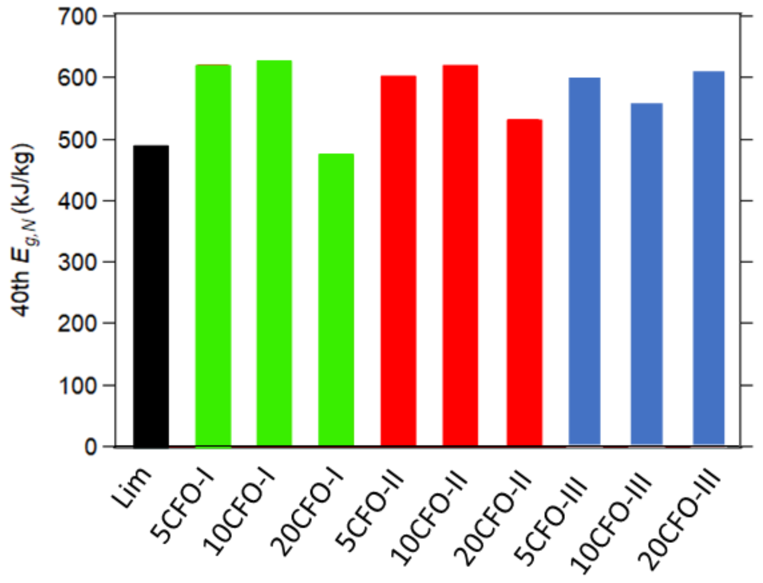

Fig. S2 Energy storage density of all samples for the 40th cycle

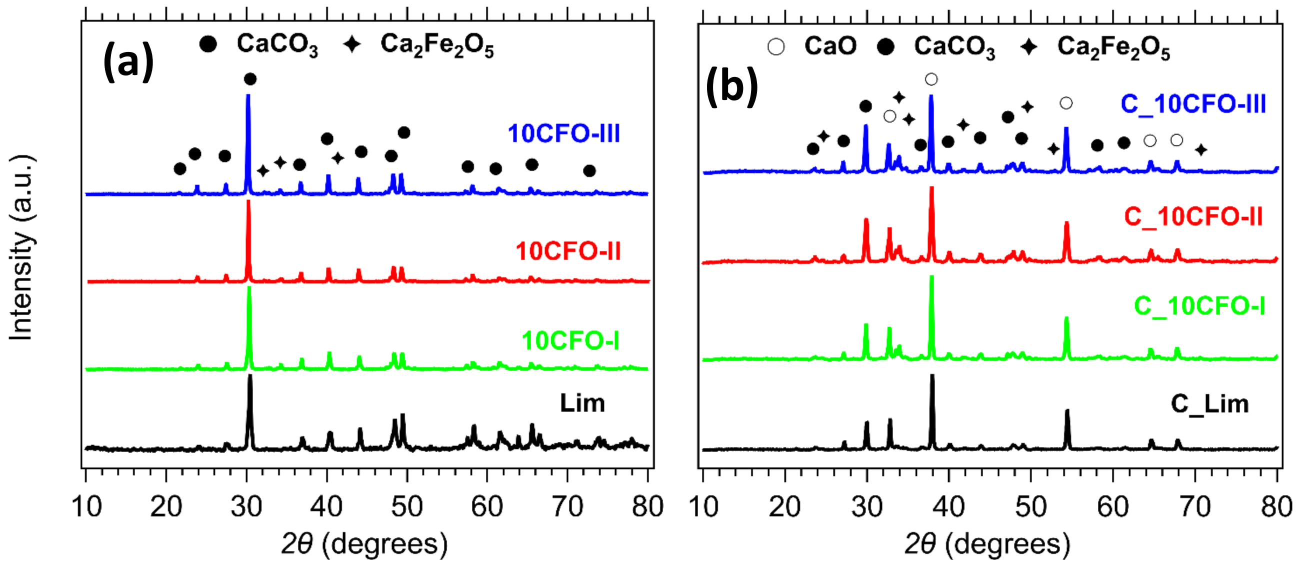

Fig. S3 XRD patterns of Lim and 5CFO samples before (a) and after cycling (b)

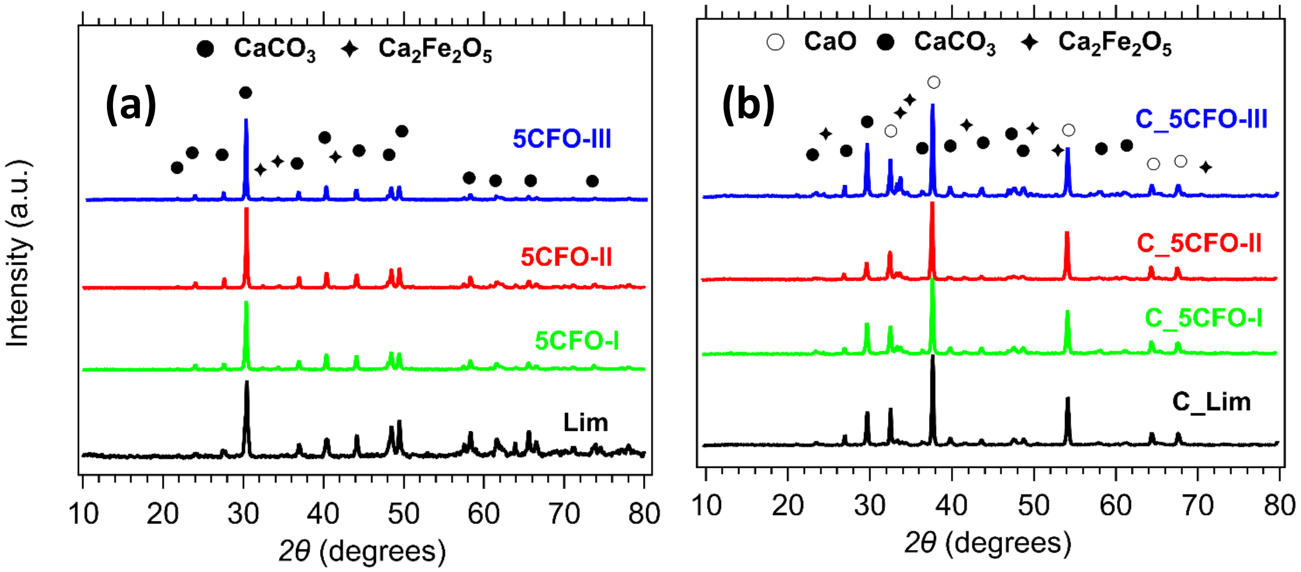

Fig. S4 XRD patterns of Lim and 10FO before (a) and after cycling (b)

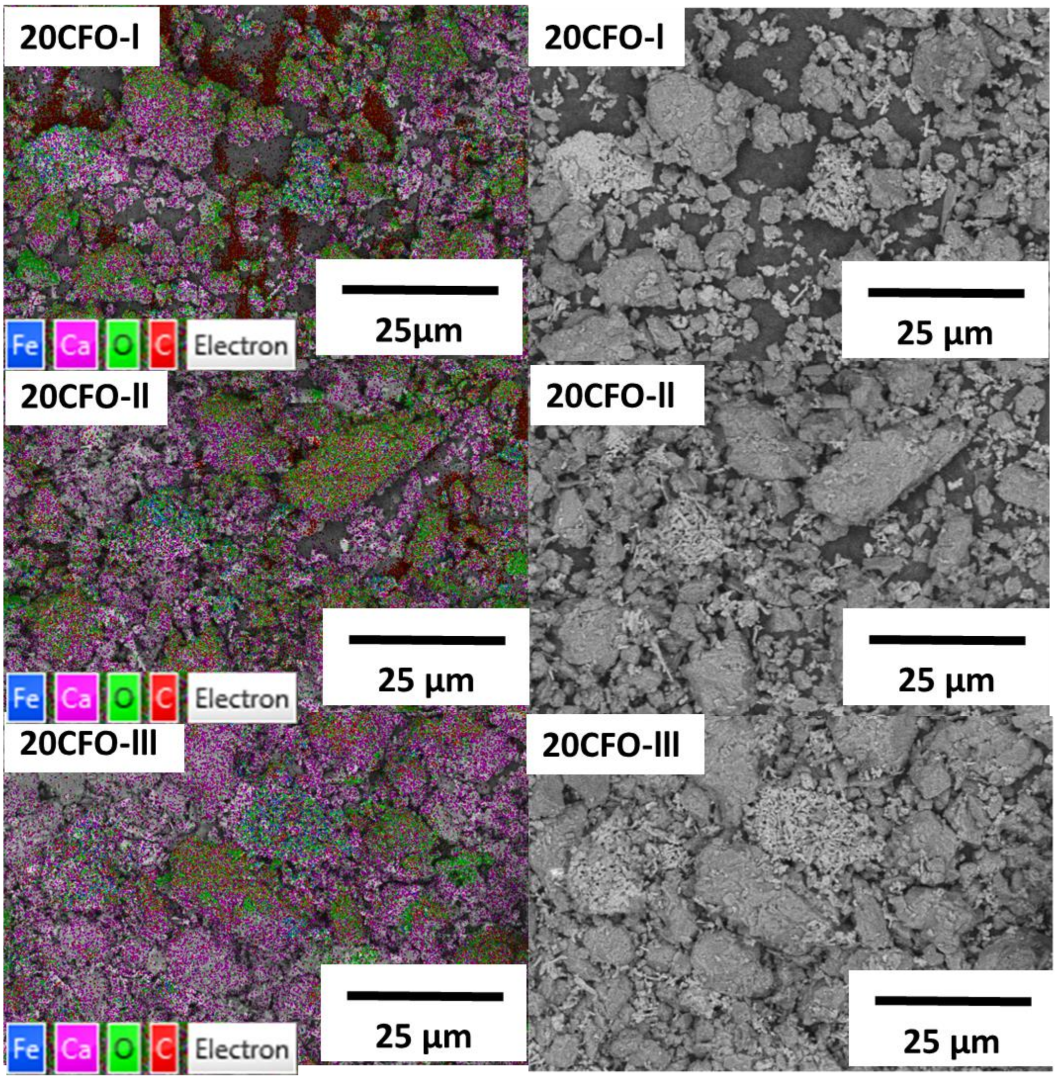

Fig. S5 EDS mapping of 20CFO-I, 20CFO-II and 20CFO-III samples as prepared

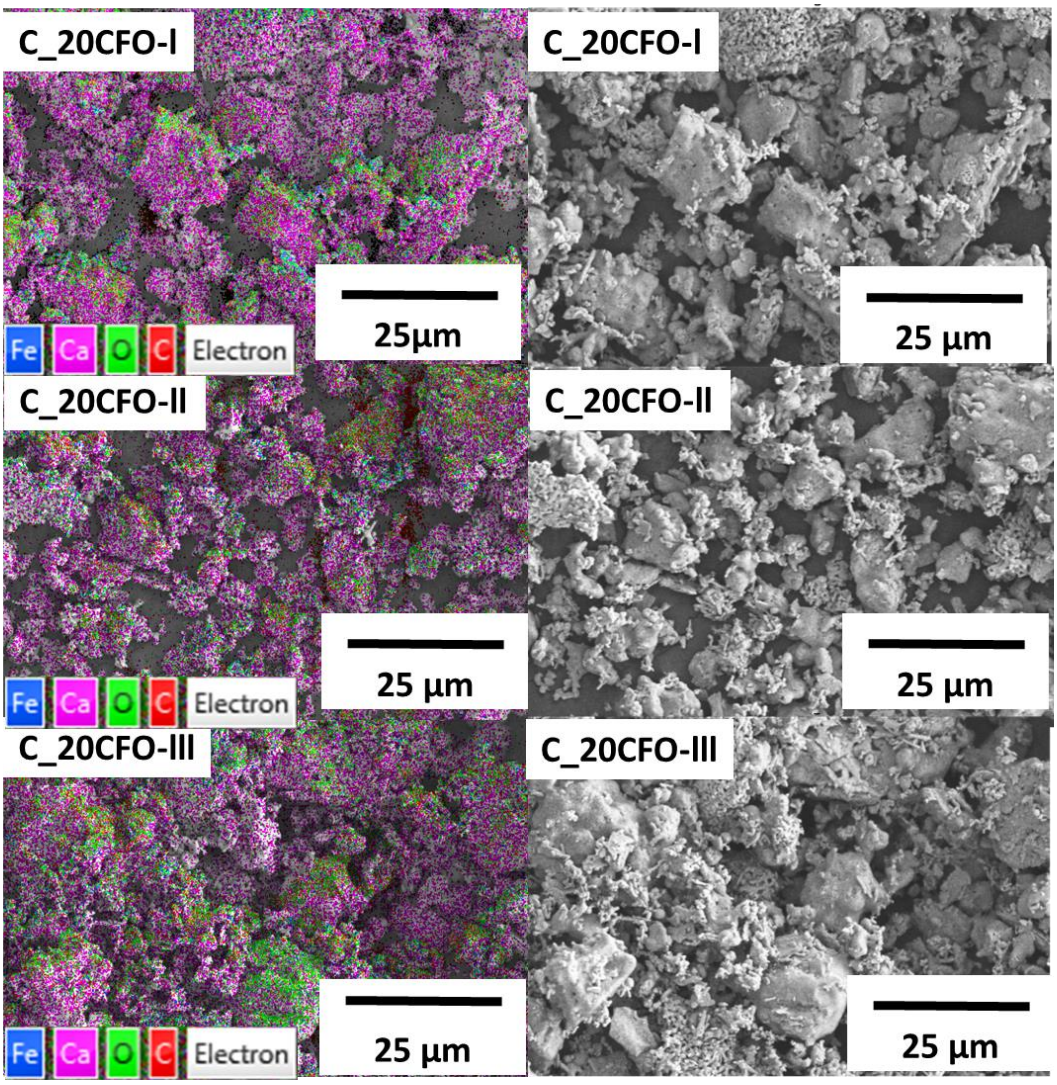

Fig. S6 EDS mapping of 20CFO-I, 20CFO-II and 20CFO-III samples after cycling

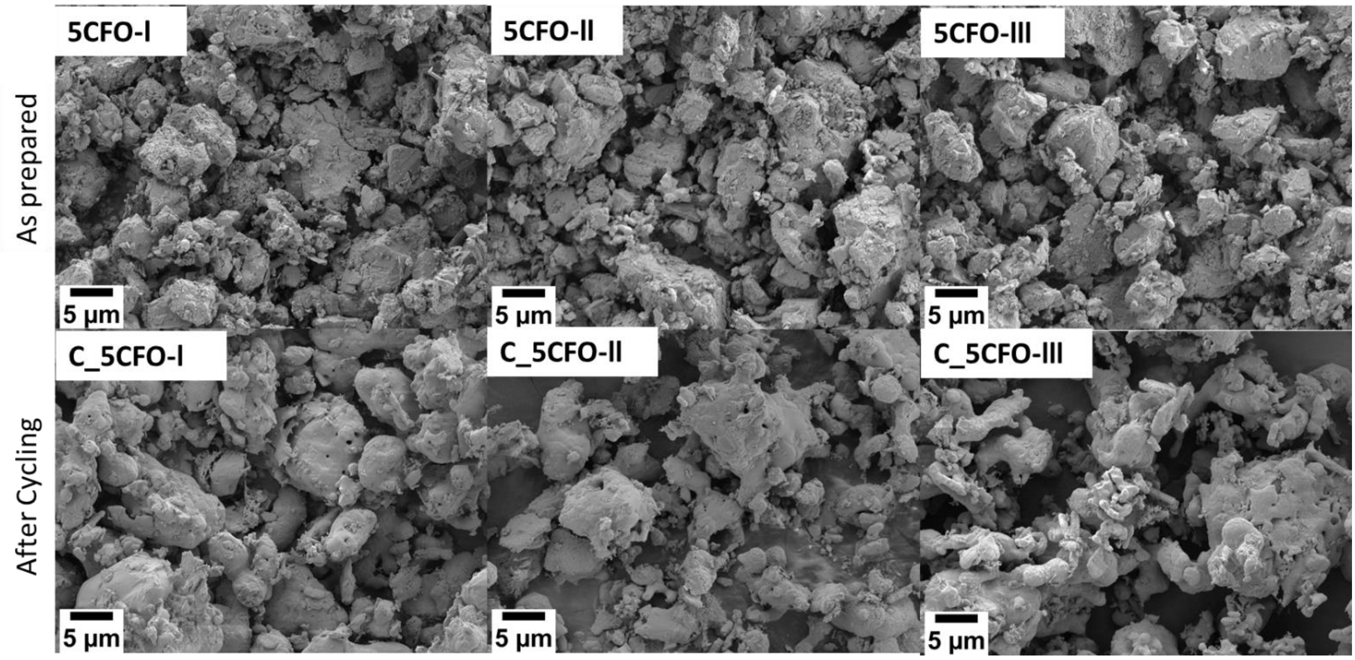

Fig. S7 SEM images of Lim and 5CFO as prepared and after cycling samples; 5CFO-I, 5CFO-II, and 5CFO-III; C_5CFO-I, C_5CFO-II, and C_5CFO-III

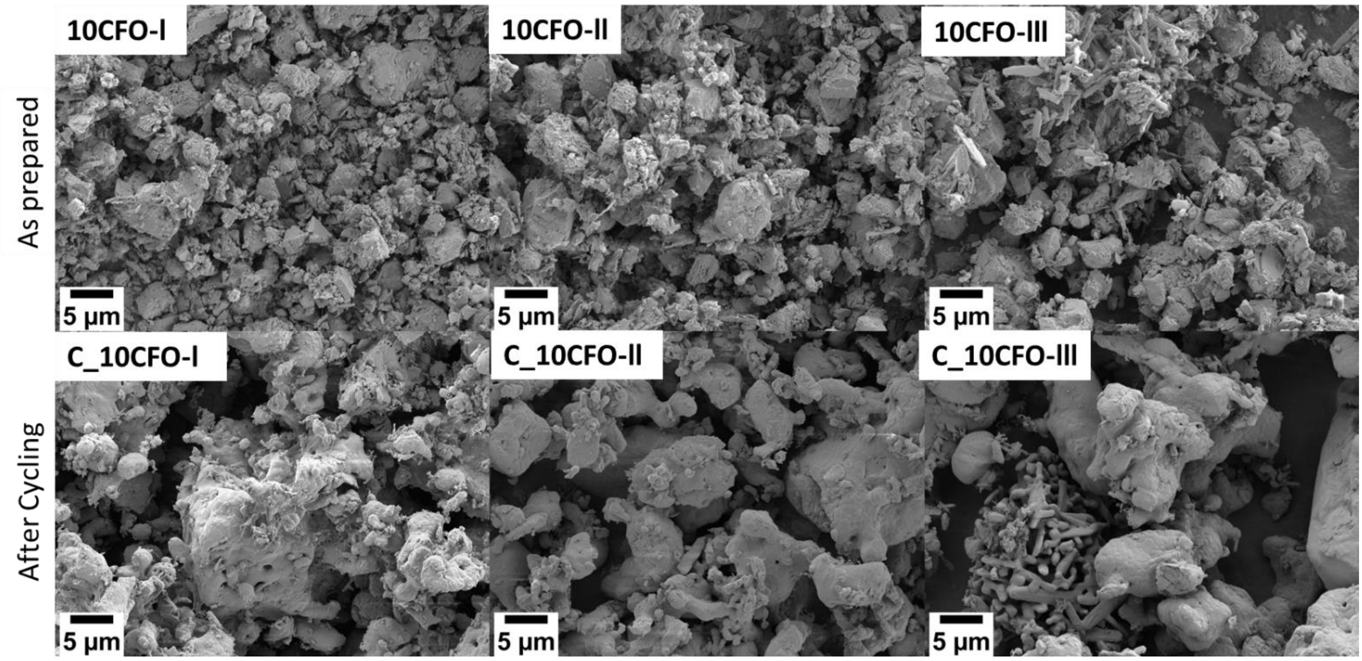

Fig. S8 SEM images of Lim and 10CFO as prepared and after cycling samples; 10CFO-I,10CFO-II, and 10CFO-III; C_10CFO-I, C_10CFO-II, and C_10CFO-III

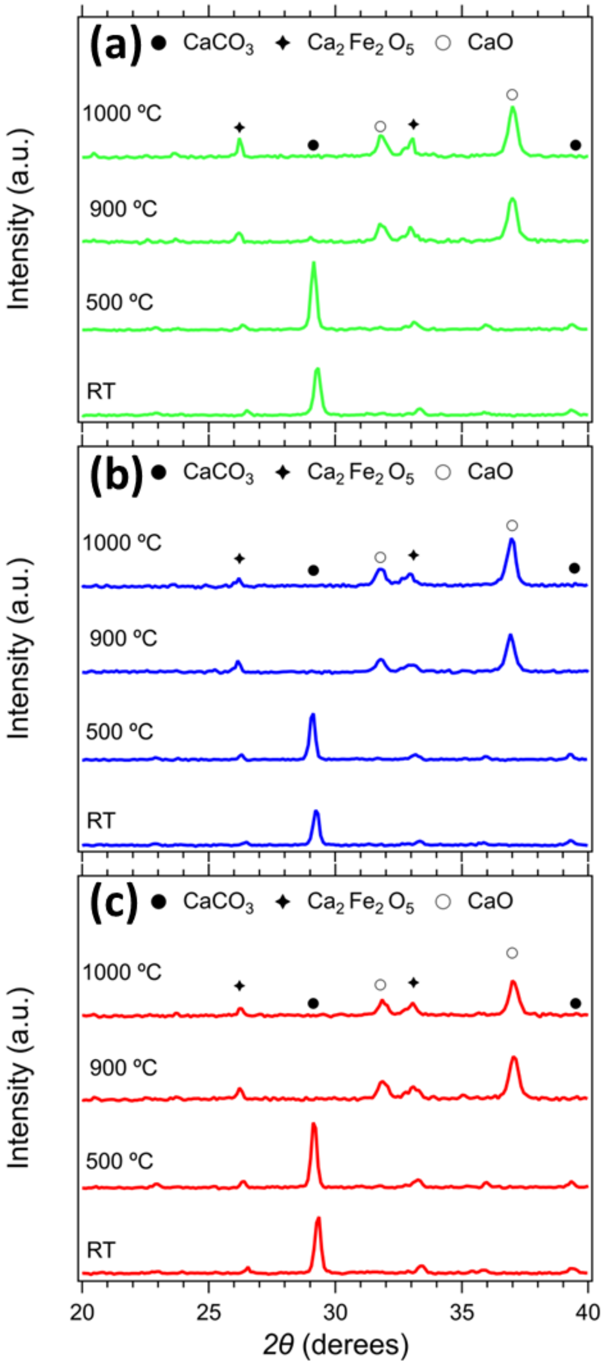

Fig. S9 In situ XRD patterns of 20CFO samples a) 20CFO-I b) 20CFO-II and c) 20CFO-III

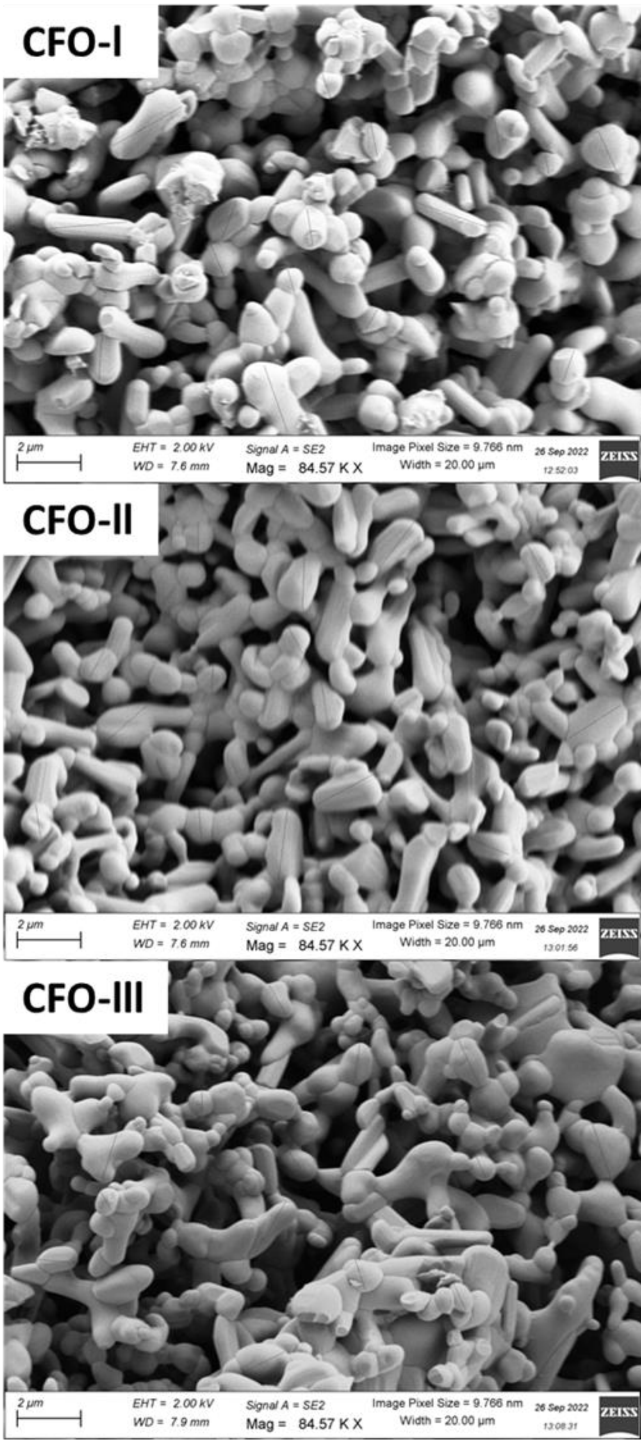

Fig. S10 SEM images of CFO-I, CFO-II and CFO-III showing lines on the particles, considered for calculation of average particle size

The standard enthalpy of reaction at a temperature T (in K) is calculated from the following equations:

$$\Delta H^o_{R,T} = \Delta H^o_{R,298.15} + \int_{298.15}^{T} \Delta c_p(T) \cdot dT \qquad (1)$$

$$\Delta H^o_{R,298.15} = \sum_{products} \Delta H^o_{f,298.15} - \sum_{reactants} \Delta H^o_{f,298.15} \qquad (2)$$

$$\Delta c_p(T) = \sum_{products} c_p(T) - \sum_{reactants} c_p(T) \qquad (3)$$

Standard enthalpy of formation at 298.15 K values, $\Delta H^o_{f,298.15}$, were taken from NIST data base for CO_2 and CaO and for $CaCO_3$ from (Wagman, D. D.; Evans, W. H.; Parker, V. B.; Schumm, R. H.; Halow, I.; Bailey, S. M.; Churney, K. L.; Nuttall, R. L. The NBS Tables of Chemical Thermodynamic Properties: Selected Values for Inorganic and C1 and C2 Organic Substances in Sl Units, J. Phys. Chem. Ref. Data, 1982, 11, Supplement 2)

From Eq. 2 $\quad \Delta H^o_{R,298.15} = -634.92 - 393.51 + 1206.92 = +178.5\ kJ/mol$

The equation used by your student, ΔH(850°C) = ΔH° + Cp(T2 - T1), is wrong because your student used only the c_p of CaCO3 and not the Δc_p as in Eq. 3. I also used temperature dependent c_p values from NIST for CaO and for $CaCO_3$ from (Gary K. Jacobs, Derrill M. Kerrick, and Kenneth M. Krupka, The High-Temperature Heat Capacity of Natural Calcite ($CaCO_3$), Phys Chem Minerals (1981) 7:55 59).

Actually, since Δc_p is negative the enthalpy of reaction decreases with temperature as shown in the following Table.

Standard enthalpy of reaction at different temperatures, for the reaction $CaCO_3$ -> CaO + CO_2

t in °C	$\Delta H^o_{R,T}$ (kJ/ mol)
25	178.5
100	178.1
300	176.3
400	174.9
500	173.3
650	170.3
800	167.2
850	166.1
860	165.9
869	165.7
890	165.3
920	164.6

References

1) A. Khosa and C. Y. Zhao, Solar Energy, 2019, 188, 619-630.

2) H. Sun, Y. Li, X. Yan, Z. Wang and W. Liu, Energy Conversion and Management, 2020, 222, 113222.

3) K. T. Møller, T. D. Humphries, A. Berger, M. Paskevicius and C. E. Buckley, Chemical Engineering Journal Advances, 2021, 8, 100168.

4) Antzara, E. Heracleous and A. A. Lemonidou, Applied Energy, 2015, 156, 331-343.

5) K. T. Møller, A. Berger, M. Paskevicius and C. E. Buckley, Journal of Alloys and Compounds, 2022, 891, 161954.

6) M. Benitez-Guerrero, J. M. Valverde, P. E. Sanchez-Jimenez, A. Perejon and L. A. Perez-Maqueda, Chemical Engineering Journal, 2018, 334, 2343-2355.

7) Y. Zhou, Z. Zhou, L. Liu, X. She, R. Xu, J. Sun and M. Xu, Energy & Fuels, 2021, 35, 18778-18788.

8) K. T. Møller, A. Ibrahim, C. E. Buckley and M. Paskevicius, Journal of Materials Chemistry A, 2020, 8, 9646-9653.

9) X. Chen, X. Jin, Z. Liu, X. Ling and Y. Wang, Energy, 2018, 155, 128-138.

10) T. Richardson, R. K. Vijayaraghavan, P. McNally and M. V. Sofianos, Available at SSRN 4156637.
